# Selections—Kerosene Oil, Etc.

**Published:** 1872-11

**Authors:** 


### SELECTIONS.

### KEROSENE OIL IN CHRONIC RHEUMATISM.

W. G. KEMP, L. R. C. P., London, M. R. C. S. England, Wellington, New Zealand, writes to the British Medical Journal:

Several cases have lately come under my notice in which the internal administration of Kerosene has had the most decided and marked effect in Chronic Rheumatism. I am at a loss how to explain its action; but the fact that it has completely cured several cases will be seen from the following extracts:

CASE I.—J. H., aged 46, sailor, had suffered from chronic rheumatism for twenty years. He never had an acute attack. He used to have agonizing twinges in his feet and legs; he compared the pain to that which would be produced by the skin being seized with a pair of tweezers and forcibly pulled. The attacks would come on about every ten minutes, and often last for three or four days and nights. He had been under medical treatment many times, but had never had more than temporary relief. He began to feel the interval between the attacks lengthen in six weeks after commencing kerosene; and in less than three months the pains had entirely left him. After discontinuing it for some time, the pain returned slightly, but was always cured by two or three doses. A teaspoonful was taken in a wineglassful of water every other night. The kerosene produced no unpleasant symptoms, no loss of appetite, no effect upon the bowels or kidneys.

CASE II.—D. W., aged 59, a gardener, had suffered from chronic rheumatism for twenty years, being affected in different parts of his body at different times. About two years ago he had a severe attack in his back and limbs. He received no benefit from medical treatment. After taking kerosene for ten days he began to feel great relief, and in less than a month was sufficiently cured to resume his occupation. He has never had any attack since. No unpleasant symptoms arose from taking it; there was no effect upon the bowels or kidneys. The odor of the kerosene was perceptible in the urine.

CASE III.—P. A. D., aged 50, a sailor, had suffered from chronic rheumatism for four years, being affected chiefly in the legs, arms and back. He had great difficulty in rising up and sitting down. He received no relief or benefit from medical treatment; the symptoms always returned after leaving it off. He began to feel great relief after taking kerosene for about a month, and was sufficiently well in three months to leave it off. He was obliged to commence taking it again a few months afterwards as he found the pains returning. No unpleasant symptoms arose from taking it. The odor was perceptible in the perspiration. The good effects were not so apparent in this case, owing to the patient being of a thoroughly broken-down constitution, and in reduced circumstances, so that he was unable to take as much care of himself as was necessary. He, however, found more relief from the kerosene than from anything else, and does so up to the present time.

CASE IV.—J. W., aged 36, a grocer, had suffered from constant pains in his back and legs, for twelve months, frequently being obliged to stay from work for several days together. For the last two months he had not been able to stoop down to tie his shoes. He received no permanent benefit from medical treatment. After taking three doses of kerosene he felt considerable relief, and is now, after taking it for a fortnight, able to walk with very little pain, and can stoop with ease. The kerosene caused slight diaphoresis, and acted as a stimulant and tonic. He says that he felt exhilarated after each time of taking it, and had more desire for food.

CASE V.—Mrs. B., aged 56, suffered from rheumatism in the arms and hands for a number of years. She had been under medical treatment several times, but never was cured. She took kerosene for less than a month, and was materially benefitted, and in about three months was perfectly well. She occasionally feels some return of pain; and after one or two doses of the oil it again leaves. She speaks of a decided tonic effect upon the system, becoming stronger and better after taking it.

CASE VI.—Mrs. C., aged 32, had suffered from rheumatism for about six years, chiefly in her hands, being often obliged to do no work for days together. She had generally been obliged to go to the north of New Zealand during the winter. Medical treatment was of no avail. She took kerosene for about six weeks, and being decidedly better, left it off. The pains returned after a few months; and after taking a few doses of kerosene, she "turned against it so" that she refused to take any more. She became worse, however, and took it again. After a short time she felt decidedly relieved, though she is at times liable to a return of the pains.

There is another case, the particulars of which I am unable to get, owing to the patient having left for another province.

Although kerosene cannot be called a specific for rheumatism, I think that the cases here cited are quite sufficient to induce medical men to give it a fair trial. I am unable to find any unpleasant symptom caused by taking kerosene. The great objections with many people to taking it are the unpleasant taste and smell. Some have taken it in water or milk; but I have lately heard a patient say he could take it best with salt; a pinch of salt being put into the mouth and allowed to dissolve, and the oil then swallowed, mixed with about its bulk of water. I am not aware of the remedy having ever been used internally; but I trust some medical man will be found who will give it a trial, and record the results of their cases. Externally, it is of great use in cases of burns, whether severe or slight; it seems to relieve pain more than any other application, especially if resorted to as soon as the injury is received. I have known cases of severe burn to heal up rapidly under its use alone.

